# Navigating whiteness: affective relational intensities of non-clinical psychosocial support by and for culturally and linguistically diverse people

**DOI:** 10.3389/fsoc.2024.1282938

**Published:** 2024-02-16

**Authors:** Karime Mescouto, Rebecca E. Olson, Stefanie Plage, Asma Zulfiqar, Jenny Setchell, Tinashe Dune, Sameera Suleman, Drew Cummins, Rita Prasad-Ildes, Nathalia Costa

**Affiliations:** ^1^RECOVER Injury Research Centre, The University of Queensland, Brisbane, QLD, Australia; ^2^School of Social Science, The University of Queensland, Brisbane, QLD, Australia; ^3^Institute for Social Science Research, The University of Queensland, Brisbane, QLD, Australia; ^4^School of Health and Rehabilitation Sciences, The University of Queensland, Brisbane, QLD, Australia; ^5^Institute for Urban Indigenous Health, Brisbane, QLD, Australia; ^6^Australian College of Applied Psychology, Sydney, NSW, Australia; ^7^World Wellness Group, Brisbane, QLD, Australia; ^8^Sydney School of Health Sciences, The University of Sydney, Sydney, NSW, Australia

**Keywords:** mental health, culturally and linguistically diverse communities, whiteness, affective economies, psychosocial support

## Abstract

Mental health is political, with intersecting economic, cultural, racialized, and affective dimensions making up the care assemblage, signalling how care is conceptualised and who is deserving of care. In this article, we examine emotions circulating in a non-clinical psychosocial support program for culturally and linguistically diverse people experiencing mental ill-health, foregrounding the relations between culture, race, economy, and assumptions underpinning understandings of care. The mental health program under study offers psychosocial support for culturally and linguistically diverse people to manage life challenges and mental ill-health exacerbated by navigating the complexities of Australia’s health and social care systems. We draw on interviews with clients, staff, and providers of intersecting services, employing Ahmed’s concept of affective economies and Savreemootoo’s concept of navigating whiteness to examine the care assemblage within interview transcripts. We provide insight into affective intensities such as hate, anger, and indifference embedded in white Anglo-centric services, positioning culturally and linguistically diverse people on the margins of care. Non-clinical psychosocial support programs can counter such affective intensities by training and employing multicultural peer support workers—people with lived experience—prioritising relational and place-based approaches to care and supporting and providing clients with relevant skills to navigate an Anglo-centric care system. However, this support is filled with affective tensions: (com)passion, frustration and fatigue circulate and clash due to the scarcity of resources, further signalling what type of care (and with/for whom) is prioritised within Australian relations of care.

## 1 Introduction

Emotions connect the individual to the social (Jasper, 2018; Patulny et al., 2019). In the context of mental health, they are central to experiences of illness (Thoits, 2010), but also to the social divisions that imbue formal and informal (mental) health care work. In higher status health care professions (e.g., doctors), emotions are often backgrounded—positioned as threats to clinical reasoning (McNaughton, 2013; Olson et al., 2022)—or sequestered to the work of lower paid health care workers, such as nurses (DiBenigno and Kellogg, 2014) or social workers (Dragojlovic and Broom, 2018). In western approaches to mental health care specifically, emotions are foregrounded by higher status health professionals, but not contextualised in relation to sociocultural factors (Davies, 2015). The care work involved in public or private settings is typically reserved for women, especially women from racially marginalised backgrounds (Glenn, 2010; Olson, 2022), with several studies revealing the compounding effects of racism and emotional labour on health care workers (Mapedzahama et al., 2012; Cottingham et al., 2018).

Attending to (mental) health care’s affective entanglements draws attention to the taken-for-granted racial, gendered and classed inequities within health and social care, embedded within the political and ideological landscapes in which they are produced. In cancer care, for example, people with cancer can frame risk as an individual responsibility due to lifestyle choices, without considering wider relations of production and consumption connected to such choices (Plage and Olson, 2021). This framing was reflected in the affective dimensions of cancer narratives, where some types of cancers, such as lung cancer, are linked to lifestyle choices, suggesting who is implicitly cast as (un)deserving of care and funding opportunities. Note, affect is used here somewhat interchangeably with emotion, with affect referring to the embodied sensations and emotion the same embodied sensations that are assigned a label following cultural categories (Ahmed, 2015). This study examines the emotional dynamics of clients, staff and providers of intersecting services of one non-clinical psychosocial support program designed for and by culturally and linguistically diverse migrants. The non-clinical program centres on support provided by multicultural peer support workers—people with lived experience—without formal clinical training such as counselling, psychology or psychiatry. Drawing on Ahmed’s (2004) ‘affective economies’ and Savreemootoo’s (2020) ‘navigating whiteness,’ we foreground emotions in our analysis to expose the moral and political ideologies underpinning work in this space.

## 2 Background: The intersection of mental health, culture and race

Australia is one of the most culturally diverse nations, with more than half of the population either being born overseas or having a parent born overseas (Australian Bureau of Statistics, 2021a). Although the majority of migrants are from English-speaking countries, culturally and linguistically diverse groups^1^ have been increasing due to intake of voluntary migrants and refugees (Australian Bureau of Statistics, 2021b). This increase requires Australian healthcare services to be responsive to multiculturalism, that is, to negotiate a culturally safe approach as decided by care recipients, including mental health services (Dune et al., 2021). People from culturally and linguistically diverse backgrounds are among the most marginalised groups in developing countries, with a high prevalence of mental ill-health (Cross and Singh, 2012). People from culturally and linguistically diverse backgrounds, especially refugees, have high rates of post-traumatic stress disorders, depression and anxiety (Bhugra et al., 2011; Close et al., 2016; Blackmore et al., 2020; Rashki Kemmak et al., 2021), exacerbated by further social inequalities (Rashki Kemmak et al., 2021); factors such as age, gender, income, education, housing, employment, English language proficiency, race and ethnicity have a significant impact on the mental health of culturally diverse people. The increased risk of mental ill-health also clearly persists into the second generation of migrants, who are at increased risk for severe mental ill-health such as schizophrenia and other psychotic disorders (Bourque et al., 2011). These and other studies highlight the interconnectedness of cultural and social inequalities, shedding light on the danger of focussing only on cultural constructions (e.g., peoples’ views about the symptoms, aetiology, and experiences of mental ill-health) rather than also acknowledging the structural factors underpinning mental health (Brijnath and Antoniades, 2018; Burford-Rice et al., 2020; Sullivan et al., 2020).

Cultural and social factors have a significant impact on and role in the development of mental ill-health (Center for Mental Health Services and National Institute of Mental Health, 2001). For example, people from culturally and linguistically diverse backgrounds can face challenges accessing care, including mental health services (Khatri and Assefa, 2022). In terms of accessing mainstream services, there can be understandable choices made by communities to access traditional healing practices as preferred approaches at least in part because Western cultural perceptions of mental ill-health that can be interpreted as highly stigmatising (Amodeo et al., 2004). Another factor is a fear of the government and related institutions (especially for political refugees or migrants whose status is contested in the legal system), including concerns that the diagnosis would affect their visa status (Barudy, 1989). However, reduced access can also be due to how mainstream mental health services are organised, with few services attuned to the complexities of being a culturally and linguistically diverse person: the difficulty of navigating Euro-centric healthcare systems in combination with other welfare, legal, and non-governmental services, the lack of cultural sensitivity to the mental health care needs of people from outside Euro-American culture, a distain for traditional healing practices, and a consequent lack of trust between patients and healthcare providers (Mortensen, 2010; Wohler and Dantas, 2017).^2^ This lack of cultural sensitivity is seen to emerge from conceptualisations of mental health in western countries.

Studies have long criticised how mental health and mental health care are conceptualised in the global north within a universal framework. Within such a framework, mental health disorders follow a universal or global reality, with concepts and constructs applied everywhere and to everyone (Alegría and McGuire, 2003; Summerfield, 2012; Brossard and Chandler, 2022). Examples of such a universalist view of mental health can be seen in the movement ‘Global Mental Health’, put forward to guarantee anyone with mental ill-health is attended to, irrespective of race, location, or creed (Whitley, 2015), and the global adoption of the Diagnostic and Statistical Manual of Mental Disorders (DSM-5; Bredström, 2019). Seemingly progressive, in terms of reach/accessibility, both approaches are considered ethnocentric due to them having a narrow understanding of culture (Whitley, 2015; Bredström, 2019). They perpetuate views that are common in the mental health space: (1) that mental health disorders are due to disfunctions of the brain or biological imbalance, without acknowledgement of sociocultural influences (Brossard and Chandler, 2022; see Footnote 2); and (2) that culture is only considered in the context of ethnic minority groups and non-Western or non-Euro-centric cultures. Culture, and similar concepts such as cultural competency, are unintendedly used as a construct for Othering non-dominant cultures, positioning culturally and linguistically diverse people in the margins of care (Mortensen, 2010; Savreemootoo, 2020). Unlike cultural competency, cultural humility purports the need for ongoing critical self-evaluation, critique, and the ability to both examine and address power-imbalances that exist in healthcare and other settings (Tervalon and Murray-García, 1998). The marginality of people from culturally and linguistically diverse backgrounds is enhanced when culture and race intersect, with the understanding of culture often closely connected to race in public imaginaries (Riggs, 2007; Ferdinand et al., 2015). In our conceptual framework below, we outline the analytical tools used in this article to explore the affective relationalities of non-clinical psychosocial support as an emotional entanglement across culture and (in)formal care.

## 3 Conceptual framework

Emotions are inherently connected to mental health care and signal broader social, cultural, and economic agendas. According to Ahmed’s (2004, p. 119) ‘affective economies,’ “emotions *do things*” and are more than psychological constructs. She argues that tracing or exploring how emotions circulate makes it possible to see the relationships between “the psychic and the social, and between the individual and the collective.” Emotions offer a lens to explore individual-collective moralities (Plage and Olson, 2021). Ahmed’s affective economies is particularly useful in emphasising the intersection between emotions and race. For example, she explores how emotions such as hate and fear stick to racialized bodies, not because the black body is hateful or fearful for itself nor that white bodies are the authors of such hate and fear, but because these affective intensities expose historical associations that connect (or disconnect) emotions and bodies. Emotions then are a form of affective intensity that connect certain individuals while excluding or privileging others when providing care—in both formal and informal care settings (Olson, 2022).

Within the mental health care space for culturally and linguistically diverse people, emotions, race, and power intersect with culture. We define culture here as something broad: incorporating the beliefs, values, and attitudes of a certain group and integrate the materiality of culture and geopolitical processes (such as coloniality) into our approach to culture (Brossard and Chandler, 2022). The concept of culture has been mistakenly used as a synonym to explore racialized ‘others’, while considering ‘Western’ or ‘whites’ as lacking culture (Brossard and Chandler, 2022). This narrow and binary conceptualization can be found within cultural competency practices, where healthcare professionals are trained to understand ‘other cultural practices’ instead of reflexively analysing how their own culture and privilege (usually centred in white Western practices) impact the people they provide care for (Savreemootoo, 2020). In the context of mental health, for example, Brossard and Chandler (2022) describe widespread assumptions that mental illnesses are universal, imposing Euro-American psychiatric conceptualisation of mental ill-health on other cultures with differing ways of seeing self, society, and mental health. To counter such narratives, we use Savreemootoo’s (2020) concept of *navigating whiteness* to shift the focus from ‘other cultures’ to exploring how white privilege, or whiteness, impacts the mental health care provided for culturally and linguistically diverse people. According to Savreemootoo (2020, p. 2), “shifting the focus to white privilege is a crucial step in deconstructing how we view race and culture.” Situating non-clinical mental health care for culturally and linguistically diverse people around intersections of emotion, race, and culture, enables us to expose assumptions of what counts as care and who is deserving of care.

## 4 Methods

Drawing on Ahmed’s (2004) ‘affective economies’ and Savreemootoo’s (2020) ‘navigating whiteness’, we attended to participants’ interviews and observe the circulation of emotions. We discuss the assumptions underpinning moral affectivities of providing non-clinical psychosocial support for culturally and linguistically diverse people.

### 4.1 Study design, setting, recruitment, and data collection

This theory-driven thematic analysis study (Pierre, 2021; Braun and Clarke, 2022) used data from a wider program evaluation on the characteristics of specialist psychosocial mental health support programs for people from culturally and linguistically diverse backgrounds in Queensland, Australia (#2022/HE001102). The evaluated organisation has a multicultural mental health program in an urban area that offers non-clinical supports to adults with mental health and psychosocial issues from culturally and linguistically diverse backgrounds. It employs a wraparound approach to delivering a culturally tailored service. In other words, care provision is not limited to one element of need, such as depression. Instead, mental health is understood as embedded within systems and services. Thus, the program offers support where it is needed, ranging from help with navigating public transport, to relaxation interventions, to connection with and support in accessing allied legal, social and medical services. Furthermore, the service is culturally tailored, with staff members from migration, refugee, and asylum-seeking backgrounds and/or the same or similar cultural and linguistic backgrounds to clients, aiming to improve the social, emotional, mental and physical wellbeing of migrants, refugees and people seeking asylum. We use the data from this wider evaluation program to think broadly about services and approaches to mental health.

Interview participants were recruited following stakeholder mapping, completed collaboratively with key staff members at the evaluated organisation. The final sample included staff members, stakeholders (e.g., from service providers identified during the mapping exercise) and clients. Recruitment followed a purposive sampling strategy (Daniel, 2012), where we prioritised certain characteristics such as diversity in type of service used, visa status, cultural background, age, service context (e.g., face-to-face or remote modalities), length of time in Australia and length of engagement with the evaluated organisations.

We conducted a total of 37 semi-structured interviews with 53 participants between August 2022 and November 2022. Nine were conducted as group interviews, and 28 individually (see Table 1). Participants included 25 staff from the evaluated organisation, 12 stakeholders (mostly staff or volunteers from other non-government organisations supporting the same population) and 16 clients. On average, interviews lasted 45 min (ranging from 16 to 83 min). Interviews were facilitated by members of the research team (AZ, SP or RO). Adhering to participants’ preferences, interviews were facilitated in a mix of modes: face-to-face, video conference and telephone. We used a telephone-based professional translation service to support communication in 8 clients’ interviews. Another 3 client interviews were facilitated in the participant’s preferred language relying on our service providers’ staff and volunteers. The interviews were conducted in the following languages: English, Spanish, Turkish, Vietnamese, Arabic, Indonesian, Amharic, Farsi, and Tamil. Although valuable, we recognise that the use of interpreters in qualitative interviewing raises additional complexities, in particular when issues are discussed that could be perceived as sensitive. We encountered some challenging dynamics brought into the data collection by using interpreters, for example interpreters pushing for clear and direct answers, where the interviewers sought to encourage open-ended reflection, or when carefully rephrased prompts to elicit more profound answers, were simplified or restated in terms of the original questions. We attempted to mitigate these issues, wherever possible by briefing interpreters prior to the interviews. Team discussions and reflections on the work with interpreters on this study, also helped to direct our thinking towards a less visible dimension of marginalisation in healthcare, given that we relied on the same interpreting service that is commonly utilised to facilitate health care encounters. In other words, our experiences as researchers relying on interpreting services to engage study participants in our research, sensitised us to issues that unfold in care encounters.

**Table 1:** Individual and group interviews.

	Individual interviews	Group interviews	Total
Staff	6 interviews (*n* = 6)	6 interviews (*n* = 19)	12 interviews (*n* = 25)
Stakeholders	8 interviews (*n* = 8)	2 interviews (*n* = 4)	12 interviews (*n* = 12)
Clients	14 interviews (*n* = 14)	1 interview (*n* = 2)	15 interviews (*n* = 16)
	*n* = 28	*n* = 25	*n* = 53

### 4.2 Data analysis

Interviews were recorded, transcribed, and deidentified for analysis. Data analysis was performed iteratively throughout the study. First, members of the research team (SP, RO, NC, JS) conducted an initial theory-driven analysis, guided by principles of post-qualitative inquiry that position theory as method (Aagaard, 2021; Pierre, 2021), using the work of Ahmed (2004) and Savreemootoo (2020). Following the principles of post-qualitative inquiry (Pierre, 2021) and the increasing calls for involving stakeholders in research (National Health and Medical Research Council, 2018), the team contributed to analysis during formal researcher-service meetings, when reporting on the analysis (this was done with the approval of the service who commissioned the evaluation), and when refining the analysis for write up. Therefore, the choice of concepts was also indirectly constructed and informed through formal and informal discussions, meetings, and planning with people from the evaluated service (SS, RPI, DC).

Second, the first author (KM) analysed the interviews separate to the broader research to become familiar with the dataset following principles of thematic analysis such as considering researcher subjectivity, active construction of themes and codes, and acknowledging that data analysis is underpinned by theoretical assumptions (Braun and Clarke, 2022). The first author then progressed to a more theory-driven analysis drawing on the concepts of ‘affective economies’ and ‘navigating whiteness’ from the preliminary work of the research team to add further insight to her thematic analysis. The findings produced in this analytical stage were subsequently refined by RO and KM with iterative input from the listed authors.

Listed authors are members of the research team or service that was collaboratively evaluated. We acknowledge that the findings report and critique of the evaluated service might have been—unconsciously—softened due to the presence of the co-authorship. However, we believe this softening did not impact the main messages and interpretation of the data as the data interpretation was constructed through various processes that included all listed authors. Authors represent diverse cultural, linguistic and disciplinary backgrounds: sociology, psychology, physiotherapy, social work and occupational therapy.

In the section that follows, findings are presented supported by data display. Following data displays, in lieu of pseudonyms, we have provided the interviewee category (staff, client or stakeholder).

## 5 Findings

In this section, we trace the circulation of affective intensities within the provision of non-clinical psychosocial support for culturally and linguistically diverse people. We posit that these affective intensities are relational, circulating and intersecting within political, ideological and racialized contexts to expose assumptions about what counts and who is deserving of care.

### 5.1 The affective politics of hate and anger in providing care

Staff supporting people from culturally and linguistically diverse backgrounds elicited different emotions during interviews. Hate and anger were identified and considered strong emotional signifiers connected to who is entitled to care and support. For example, one stakeholder interviewee from a service provider, working in the community service sector, reflected on her experience with a client who her organisation and the evaluated organisation supported:

I found out then [that both organisations provided mental health referrals and citizenship support]. And **that’s one thing I really hate**, is doing work that someone else is already helping someone with, because it’s such a waste of all of our time when we have limited funding, and it’s kind of like **I hate that**… … So, I realised that we’d been double upping quite a bit. (Stakeholder interview 7)

This interviewee states, she “really hates” when clients double-up on services. But, hate can be considered a strong emotional signifier in this context. Does it relate to a sense of betrayal? The client’s perceived ingratitude for all her work? Although not explicitly related to a cultural context, connecting this affect to its political and ideological context seems to underscore a moral dimension to the hate—even if hate was not necessarily what the interviewee really felt.

The services aim to address broader needs of culturally and linguistically diverse populations, who usually need more support due to their psychosocial needs. By implying that extra care is wasteful and by connecting to hate, the stakeholder might be reinforcing power dimensions that position culturally and linguistically diverse people as less entitled to extra care and support. It also suggests that this worker feels an affective inequality—the benefit to her/her organisation for her work is potentially threatened by the double-up on services.

The above exchange also occurs outside of a fee-for-service context, and rather than a monetary transaction, an affective exchange of gratitude and loyalty is expected from the care recipient. This gratitude and loyalty were also commonly expressed by clients, who sometimes would not voice their challenges with certain organisations because—as mentioned by one client interviewee—“… it’s a service that is helping me” (Client interview 7). Consider this staff interviewee’s reflection:

for them to live in Australia, it requires for you just to work, work, and work more. And now they start facing that age where they’re not able to do that work anymore, so they feel worthless. They feel that they are depending of so many people now, and that takes a lot of their mental health and takes a toll on them. And eventually it’s taking a toll on the family because they’re trying to help, but they’re also busy with their lives. And then it’s want to feel that they’re providing to the community and being worth of something (Staff interview 11).

In another interview (Client interview 1), a client from Croatia who does volunteer work with people from Yugoslavia described the experiences and emotions involved when supporting a Bosnian client who has also engaged with the evaluated organisation:

what [his doctor] was **really angry** about was that … [The doctor] had referred this gentleman to [a] diabetes [plan] and the gentleman hadn’t attended, but the reality was that that particular attendance was going to be of no use because this elderly person does not cook, he eats from soup kitchens or from people’s places. And so it was really challenging. Because my intervention was “Doctor, I just need to explain to you why he hasn’t done what he’s done.” And then **the doctor yelled at me or shouted at me** and I thought, “Whoa, this can’t happen or continue.” So, I did a little bit of searching and I found [the evaluated organisation]…

Here, the doctor’s anger ‘sticks’ (Ahmed, 2004) to the client from Bosnia. It reflects the power difference and hierarchy between the doctor and the patient—the doctor seemed angry with the patient for not complying with the ‘care’ being offered. Again, the doctor’s anger and disproportionate reaction leads to the question of why such a reaction happened. Race,^3^ socioeconomic and migration status might intersect here. Diabetes programs usually contain lifestyle change strategies such as better nutrition to improve the body’s sugar level. However, the elderly patient eats from soup kitchens or people’s places, indicating a lack of autonomy and economic resources to control what he consumes. Whiteness as a system embedded in healthcare is bound to a liberal project (Mayes, 2020), where compliance and adherence to treatment are assumed to be the norm, and expectations follow a pattern of an idealised white, middle-class patient with the resources to successfully achieve the lifestyle changes proposed by healthcare plans. The affective display of anger might make visible—even if in subtle ways—institutionalised forms of oppression along racial and class divisions.

Hate and anger thus circulated in healthcare services and non-clinical relationships, not as a conscious endeavour connected to “hating” something or someone, but which nevertheless moved the interaction in certain directions. Like Ahmed’s (2004) consideration that fear can be an instrument of power moving racialized bodies to become fearsome, hate and anger can be an instrument and effect of power, showing how culturally and linguistically diverse bodies are treated as less entitled to care.

### 5.2 Indifference towards difference

The affective exchanges circulating in the interviews were not limited to hate and anger. Indifference was similarly present and reinforced specific power dimensions, positioning culturally and linguistically diverse people in the margins of care. In contrast to stronger signifiers such as hate and anger, indifference was less obvious, but the work of exposing such indifference reveals hidden inequalities that otherwise might go unnoticed. The indifference indicates that the default culture within Australia’s health and social care system is Anglo-centric, limiting the capacity to recognise and act on cultural diversity.

Indifference was present even when services were targeted to culturally and linguistically diverse communities, entangled with other emotional states (e.g., exhaustion), as explained by a client below:

**I find it really exhausting, to be quite honest.** Because one of the things that is really obvious to me is that any services that exist in this country, where their focus is people from other cultural backgrounds, they still tend to offer those **services through what I call a Euro- or an Anglo-centric perspective and not think outside the square** (Client interview 1).

Here, Australian health services are said to be indifferent to non-dominant (non-Euro, non-Anglo-centric, Non-White) cultures. Indifference and exhaustion expose assumptions underpinning health services: assumptions of the universalism of White and English-speaking conceptualisations of self, health and care. The indifference to non-dominant cultures reinforces Savreemootoo’s (2020) call for reflexivity regarding Whiteness to make visible the pervasive power imbalances of white privilege and white culture in how care is structured and provided. Such low-intensity emotional experiences—indifference—are therefore entangled with certain constructions of race and culture within healthcare. To counterbalance this indifference, ways to support clients outside Westernist mental health care practice (and therefore think “outside the square”) could be to assist with the provision of childcare, connect with electrical or plumbing services, support smoking ceremonies to appease ghosts, and other practices that attend to clients’ broader sociocultural needs.

Indifference was also present in mental health services’ unwillingness to adapt to the specific needs of culturally and linguistically diverse people. For example, participants noted that it was common for mainstream mental health services to either take a long time to adapt their services (e.g., arrange an interpreter, share clients’ information with support workers) or even refuse to adapt their services altogether. An example can be seen below where a staff member highlights how healthcare providers would not usually connect their clients with specialised services:

I can see sometimes psychologists that are very centred in their therapy and they sometimes **don’t want to do or take those steps forward about how to advocate or make things easier for the client with just the home call to organisation to connect them or anything like that.** So, we do that. (Staff interview 5)

The non-clinical support provided by the evaluated organisation was a way to resist the indifference from certain services and advocate for clients. A phone call that would “make things easier for the client” (Staff interview 5) and provide them with further support was considered outside the scope of usual white Euro-centric care practices and assumptions—designed for those who know how to navigate the Australian health system. Psychology as a white institution leads psychologists to be indifferent towards difference by adopting a colour and cultural blindness belief that all peoples’ experiences in healthcare are the same without the need for further support or adjustments. A similar argument has been raised with the nursing emphasis on empathy that leads to a “colour-blind” attitude towards the disproportionate emotional labour faced by nurses of colour (Cottingham et al., 2018).

Like the affective intensities of hate and anger, indifference does something (Ahmed, 2004); it sticks to clients’ bodies and conserves power by distancing the care provided to them and white Australians. Indifference contains culturally and linguistically diverse bodies within certain spaces (Ahmed, 2004)—“making things easier for clients” is deemed too hard or complex and, therefore, out of scope of mainstream services. Indifference widens inequalities.

### 5.3 Embracing and celebrating differences through trust, safety and (com)passion

The staff of the evaluated service—largely women from culturally and linguistically diverse backgrounds—work to unstick anger, hate and indifference from their clients. Embracing and celebrating differences are at the core of the evaluated service, where most staff members are from culturally and linguistically diverse backgrounds, especially multicultural peer support workers. Such celebration is exemplified by the circulation of trust, safety and (com)passion in the flexibility of where care is provided, the relational approach to the construct of culture, (com)passion entangled with the work, and navigation of whiteness. The below examples shed light on what Brossard and Chandler (2022, p. 116) considered a big dilemma when considering culture in mental health: “to consider, at the same time, that humans are equal in principle, while acknowledging their difference, without reducing them to these differences nor reifying these differences.”

Embracing and celebrating differences meant that non-clinical psychosocial support was flexible regarding the form and setting where care was provided. For example, a wellbeing support coordinator described the importance of space to the affective dimensions of care:

that’s where we are different, but also **we go to the client home**. This is a big thing for our client to **trust us, to invite us to their home**. And I think we do that really well in terms of that changes everything, I think, for CALD [culturally and linguistically diverse] client. Because a lot of them don’t drive or they don’t have anyone to drive them. Some of them haven’t been out of the house for a very long time. They don’t know how to use public transport. So, we can start there, but **it doesn’t mean that [the client] gets stuck in there**. (Staff interview 4)

Going into clients’ homes requires a sense of safety and trust between staff and clients, while also transcending the power-affective entanglements of a clinic and clinical interaction.

Clients also appreciated the power-affective entanglements and importance of space in clinical interactions:

Well, I was just too happy because they come and sit in my home and personal talk. That’s what I’m really happy about. They just sitting down. And when I offer them food already, **we eat from the same table, which I was really happy, that they could know and feel and see for themself what I’ve been going through**. (Client interview 10)

Power is symbolically rebalanced when people share food at the same table. Such affective entanglements do not only stick to bodies but to things. Trust and safety stick to the material object of a table, adding a further layer of socio-materiality to the power-affective entanglements. Sharing their home life and having a shared feeling of what they have been going through with a care provider is translated into alleviated sorrow/frustration and elevated contentment. Trust, safety, and contentment are central to the affective dimensions of care (Robinson, 2016). The location’s affective significance may help to start a process of shedding emotions that have typically been stuck to refugees, people seeking asylum and other immigrants in Australia’s ideological landscape (Martin, 2021a,b).

Embracing and celebrating differences goes beyond language. Cultural understandings and exchanges are not simply mediated by language, but relational, which requires establishing trust and safety. For example, culturally and linguistically diverse people can be marginalised for their cultural beliefs. Multicultural peer support workers have an embodied practice that embraces, rather than marginalises such difference. This relational approach was described by an interviewee with a leadership position below:

often in health services, we just keep banging on about language, “Oh, we’ll get an interpreter and we’ll be fine”. **But it’s so much more than that. And I think that’s where our peer support workers play such a vital role for that engagement. Yeah, the cultural sector, but the engagement, so that people can be themselves, they can say the stuff that they leave at the door when they see someone else**. They might be doing all sorts of cultural practices that to other people will sound crazy, like “Oh, do they believe in that?”. But they can say all of that because someone’s in the room who understands that, who’s not going to put them down for it. (Staff interview 3)

Having someone from a similar cultural background might allow clients to be themselves with seemingly little fear of judgement, without the need to leave “the stuff… at the door”. Multicultural peer support workers embrace shared differences by embodying differences and helping fear get unstuck from culturally and linguistically diverse bodies.

The affective relationship between multicultural peer support workers and clients is more than transactional; it is relational. It is not just any support worker, but one that the client connects with interpersonally and also culturally and linguistically, if the client wishes. However, some clients feared that being supported by someone from the same community might have exposed them to stigma. In addition, being from a similar cultural background might not be enough for a more relational interaction, as some communities are very conflictual, with considerable class and gender differences and political divides. Thus, the connection between clients and peer support workers needs careful consideration and flexibility for adjustments. In the peer support relationship greater emphasis was placed on a shared experience of migration and settlement in Australia, rather than proximity and belonging to a cultural community.

Support workers describe their approach as strengths-based, illustrating an important shift from popular individualistic approaches towards a relational understanding of strength: To be strong, we need allies we trust. As this wellbeing support coordinator explains:

So, **strength approach… defining their strength…. That could really empower our client.** We’re thinking about making change in their life, but also **knowing what they can do, what they’re capable of**, and for us to know so we can empower them when they are on the lowest level of mental health. Because we know there’s a time when I need to remind my client, she’s a strong woman, she can do this, and her strengths are A, B, and C. Yeah, they need that reminder. We all do. But to be able to use that as a tool, a drive to make changes. Because **they are capable of making changes, but maybe they don’t have anyone who believes in them**. (Staff interview 4)

Allies connect with clients beyond language: the relationship needs a shared understanding beyond a paternalistic notion of care. Although support workers can help and support clients, they often also know their clients’ potential to thrive without them.

The engagement with clients is also filled with passion. Passion can be defined as “a strong inclination toward a specific object, activity, concept, or person that one loves, values and that has some ties to one’s identity” (Vallerand, 2015, p. 14). This form of passion circulates through stakeholders, staff members, and clients. Passion breaks the indifference towards culturally and linguistically diverse people and shapes the relationship between care provider and client. Such passion can be seen in the two quotes from a stakeholder and peer support worker below:

I do want to acknowledge the incredible work that [evaluated organisation] were already doing before they worked with us and since we have partnered together on lots of things**. They’re so incredibly passionate and they put so much time and effort into the care that they provide and the support that they provide to participants.** (Stakeholder Interview 9)

And **I’m really happy to help my community because we are all in the same page**, the same thing when you arrive here, we’re struggling with everything and I’m really happy to help my community facilitate some form, something, which is give me more happiness to help them. Yep. (Staff Interview 10)

Here, passion and contentment are entangled, with staff, clients, and intersecting organisations affectively connected.

By embracing and celebrating differences, the evaluated service assists clients in navigating whiteness (Savreemootoo, 2020). Accessing support requires an understanding of, engaging with and negotiating white culture. Service providers translate and explain the definitions and expectations of Anglo-centric mainstream care so that clients can understand and respond accordingly. The role of the multicultural peer support worker to help clients navigate whiteness, entangled with their lived experience, can be exemplified by a staff member below:

**We need a person that is there for us, that can make a cultural contact first** and say, “How are you?” You make this connection. Things that obviously a clinician would be able to do, **but psychologist cannot touch their client. But we can**.

We can say, “How are you? How have you been?” and that’s something, and this person can feel comfortable enough and, “Look, I’m going through this and this and that. So, I have no money and I’m bust. So I don’t want to talk to my community because back home is like this and this and this and that.” But then he say, “Look, we are here together. I’ve been through something. We had our lived experience.” And this lived experience is a heal support for our fellow citizen, and that’s the most important thing in this peer support work. (Staff Interview 10)

Usual formal care (exemplified by the staff member’s mention of a psychologist) “cannot touch their client,” but as a multicultural support worker, they can. The word “touch” brings materiality to embracing differences, providing an embodied closeness and a trusting and safe relationship. According to Savreemootoo (2020), healthcare professionals of colour can offer insight into the intersection of white privilege and racial Otherness by bringing their own perspectives of experiencing Otherness to the workplace and eventually helping clients to navigate whiteness. A shared experience—coming from a culturally and linguistically diverse background and sometimes being a person of colour themselves—can “touch” the client and go places that a traditional, universalistic approach to care cannot.

Finally, multicultural peer support workers also assist clients to navigate whiteness by providing a sense of connection. Anglo-centric culture is characterised by individualistic accounts on health, where individuals are expected to resolve their problems on their own, whereas people from culturally and linguistically diverse backgrounds may come from collectivist societies. The discrepancy between an individualistic and collectivist culture has impacts on clients, as mentioned by the staff member below:

Connection is part of mental health wellbeing. We need that as human, naturally. But I think what makes it quite unique for our CALD [culturally and linguistically diverse] client is because **they grew up not being an individual. It’s very collective kind of upbringing.** So, when they experience both mental health and isolation, it’s almost double dose. (Staff Interview 4)

Brossard and Chandler (2022) highlight how wider structural conditions such as racism shape emotional isolation, compounded by the isolation due to dislocation, loss of networks and change in social roles migrants experience in a new country of residence (Quosh, 2011; Wells et al., 2018). Thus, multicultural peer support workers assist clients to navigate whiteness and counter oppressive structures, including individualistic notions in mental health care and life.

### 5.4 Frustration and fatigue in providing care on the margins

Providing non-clinical psychosocial care on the margins of mainstream services is entangled with frustration and fatigue. Although this type of care is characterised by the relational circulation of (com)passion mentioned previously, frustration and fatigue come into play due to the scarcity of funding and material support, a problem that is widespread (Amodeo et al., 2004; Behnia, 2007; Hassan, 2013; Chemali et al., 2017). Such provisions could allow staff to be better paid for their work, secure viability, and have more clients accessing the service as one staff member mentioned:

it’s doing really, really good work and has a lot of people now dependent on it, in a good way. But the business can die pretty quickly. I’ve got to tell you, being involved in the finances, we run very lean, very lean, and I don’t think that’s good. There’s no wastage, but we don’t pay people enough. And I’m all for hard work, but, man, it’s too skinny in terms of margins and that sort of stuff. **And we don’t claim enough money from government, we don’t back ourselves enough, because we’re big, beating hearts. We’re not fundamentally about making money. We’re fundamentally about helping people**. (Staff Interview 7)

“Helping people” and being “big, beating hearts” are portrayed as somewhat different from economic endeavours. Such a presupposition suggests affective relationships could be made without economic incentives, which positions relational emotions as outside the job roles of clinical and non-clinical workers (Glenn, 2010). This affective entanglement, the pride that one feels for doing ‘good’ and providing care for culturally and linguistically diverse people (Jasper, 2018), is almost considered to be enough, or a “help” instead of something that is intrinsically deserving sufficient material support and funding.

The funding availability for mental health services for culturally and linguistically diverse people was continuously mentioned as scarce. Such scarcity could be connected to indifference, mentioned in the previous heading, and Australia’s history of treating especially non-white migrants with fear and suspicion (Martin, 2021a,b). Indifference circulates not only within relationships and health services, but also within funding schemes and political priorities. Indifference again highlighted inequalities in mental health care. Indifference related to funding schemes resulted in a lack of funding opportunities. It came into tension with the (com)passion from the organisation’s leaders, who had to advocate and carve out funding opportunities to continue to do their work.

**every single mental health program we’ve got is because we agitated for it, not because it was actually a tender to apply for. So that’s a reality**. Unless we go and speak to ministers and go political, who then put pressure on the bureaucracy to listen to us, we wouldn’t have any of these programs funded. So, I think that’s why we do it and that’s why we need to keep shining the light. So yes, it is a pressure point and it’s difficult, **but unless we actually do it and highlight the needs beyond [City], those services are not going to get funded.** (Staff Interview 3)

Providing care on the margins for culturally and linguistically diverse people is thus accompanied by frustration and fatigue. (Com)passion comes into tension with frustration and fatigue due to funding logics informed by contingencies of care, while the scarcity of funding also fuels further (com)passion in continuing to provide the care on the margins. “Agitation” is necessary to argue that culturally and linguistically diverse people are also entitled to appropriate care.

## 6 Discussion

In this article, we traced the circulation of emotions, allowing us to see the intersections between race, culture, moral, political and economic dimensions of providing non-clinical psychosocial support for people from culturally and linguistically diverse backgrounds. Multicultural peer support workers—people with lived experience—without formal clinical training, provide the non-clinical psychosocial support. Our exploration exposed mental health care as rooted in white culture, where affective intensities such as hate, anger and indifference stick to racialized, culturally and linguistically diverse bodies. Although these affective intensities were counterbalanced by specific organisational efforts to embrace and celebrate differences, such intensities came with tensions characterised by frustration and fatigue in providing care on the margins of Australian health systems. Thus, our article contributes to calls to prioritise, investigate and expose intersections of race, culture, and moral affective economies of care (Ahmed, 2004; Plage and Olson, 2021; Olson, 2022).

Our findings suggest that affective intensities such as hate, anger, and indifference are entangled in ‘cultural blindness’. ‘Cultural blindness’ is the inability to understand inherent differences between peoples’ worldviews connected to cultural considerations (Kalyanpur, 1998; American Psychological Association, 2018), including how whiteness as a culture can impact the provision and (in)accessibility of mental health care for non-white or non-western populations (Brossard and Chandler, 2022). ‘Cultural blindness’ can thus be connected to universalist assumptions about mental health: a biomedical perspective that mental health disorders are dysfunctions of the brain that apply to everyone everywhere, which has been widely criticised (Summerfield, 2012; Brossard and Chandler, 2022). On the other hand, Brossard and Chandler (2022) present a radical relativist position which challenges universalist constructs of mental disorders altogether, highlighting how such a construct is a product and imposition of Western psychiatries—while acknowledging the diversity within psychiatric practices. The lack of white reflexivity, entangled with the concept of ‘cultural blindness’, underscores that everyone has a cultural background (Savreemootoo, 2020; Brossard and Chandler, 2022), and that culture is inherently interconnected to race, ethnicity, and nativity in the mental health space (Brown et al., 2013; Gopalkrishnan, 2018). Our study brings to the fore how affective intensities perpetuate and expose such assumptions within mental health care while also highlighting how care could be reframed when ‘cultural blindness’ is challenged by a broader understanding of culture and by people with lived experience working in a non-clinical mental health program.

The non-clinical psychosocial program we evaluated strives to make ‘culture visible’ through the circulation of (com)passion that embraces and celebrates differences. Emotions then play an important role in reimagining care, with similar notions to what Lynch (2009) called affective equality, where care is not mechanical and driven only by economic forces but also by relational forces such as solidarity. Compared to formal care, non-clinical and informal care involves more intense emotional experiences (Olson, 2022). The relational force seen by the circulation of passion and compassion in our study seemed to celebrate and reimagine differences since care was provided by people with lived experience who shared struggles and had insider experiences of navigating Australian Anglo-centric systems as non-White migrants. When formally employed, people with lived experience can support patients by building relationships based on mutuality, shared power, and respect (Shalaby and Agyapong, 2020). Culturally and linguistically diverse peer support workers can help improve health outcomes and have an important role in improving health access and equity for culturally and linguistically diverse communities (Henderson and Kendall, 2011). Although Savreemootoo (2020) emphasised the importance of people of colour in formal care (such as social work) in helping patients of colour navigate whiteness, our study adds that people with lived experience can also play this role. Navigating whiteness is not only limited to the constraints of formal care but also allows a broader consideration of care altogether: our study highlights how divisions between in/formal care reflect western practices and embodied conceptualisations of self, other and health. If we are to bypass a need for navigating whiteness, this requires transcending the current emotionally imbued moral economy of care: a relational-care-emotional constellation. Care is also about spaces and connections: going to peoples’ houses, taking a strength-based approach, and being part of a collective. Thus, the circulation of emotions reimagines the location and construction of care from a paternalistic and individualistic approach, where deficit-based strategies and expectations that people manage their condition are evident, to one that acknowledges peoples’ strengths, relationality and collectivity (Gorman et al., 2003; Xie, 2013). However, reimagining care and navigating whiteness as an organisation is fraught with affective tensions.

Passion and compassion are accompanied by frustration and fatigue due to insufficient material support and available funding. Compassion fatigue in the mental health space has been discussed extensively (Singh et al., 2020; Marshman et al., 2022), considered a stressor inherent in caregiving work due to the contact with trauma and adverse circumstances of others. This common conceptualisation puts an emotional state as a problem of individuals: healthcare workers who care too much and patients who face complex situations. However, another way of framing compassion fatigue, evident in the affective tensions presented here, references the political and economic constraints of what counts as care and who is deserving of care. In such a broader conceptualisation, emotions are relational (Denzin and Kemper, 1990; Olson et al., 2020; Plage and Olson, 2021), connected to individuals, expectations, and how care is funded. Funding schemes are also embedded in a white culture: mental health care is conceptualised within the constraints of an Anglo-centric system (Mayes, 2020), which positions culturally and linguistically diverse communities at the margins of care. The marginality is accompanied by a growing economic inequality, characteristic of a neoliberal capitalist society, which discourages (com)passion and solidarity over economic gains (Lynch et al., 2021). Yet, such marginality can also be a place of resistance, of radical possibility and openness to imagining alternatives (Hooks, 1989).

## 7 Conclusion

Affective relations defined the experiences of clients, providers and stakeholders of the evaluated non-clinical psychosocial support program designed for culturally and linguistically diverse people. Attending to these emotional entanglements revealed the political and affective economy underpinning relations beyond the service (and necessitating the service). Emotions of hate and anger stuck to people from culturally and racially marginalised backgrounds, revealing a moral and political affective economy of care—underpinned by ideologies of Anglocentric nationalism and neoliberalism—that implicitly position particular clients as less deserving of care. Although the non-clinical psychosocial support program examined here illustrates affective tensions, the choice and commitment to imagine and agitate for alternatives, exemplifies that marginality is a place beyond deprivation, beyond ‘otherness’: it is also a place of creativity and social transformation.

## Data availability statement

The datasets presented in this article are not readily available. Due to the sensitivity of the data and potential identification of participants’ identities, we will not provide the dataset upon request.

## Ethics statement

The studies involving humans were approved by the University of Queensland Human Research Ethics Committee (#2022/HE001102). The studies were conducted in accordance with the local legislation and institutional requirements. Written informed consent for participation in this study was provided by the participants’ legal guardians/next of kin. Written informed consent was obtained from the individual(s) for the publication of any potentially identifiable images or data included in this article.

## Author contributions

KM: Formal analysis, Writing – original draft, Writing – review & editing. RO: Conceptualization, Formal analysis, Funding acquisition, Investigation, Methodology, Project administration, Supervision, Writing – original draft, Writing – review & editing. SP: Conceptualization, Data curation, Formal analysis, Funding acquisition, Investigation, Methodology, Writing – review & editing. AZ: Data curation, Formal analysis, Investigation, Writing – review & editing. JS: Conceptualization, Formal analysis, Funding acquisition, Methodology, Project administration, Supervision, Writing – review & editing. TD: Conceptualization, Funding acquisition, Methodology, Writing – review & editing. SS: Writing – review & editing. DC: Writing – review & editing. RP-I: Writing – review & editing. NC: Conceptualization, Formal analysis, Funding acquisition, Methodology, Supervision, Writing – review & editing.

## Footnotes

^1^The terms Culturally and Linguistically Diverse (CALD) and Culturally and Racially Marginalised are used interchangeably in this paper to acknowledge the challenges based on language, culture and politics faced by those from backgrounds outside the white, European and English-speaking majority. By using the terminology “culturally and linguistically diverse,” we also acknowledge the embedded assumption that people from English-speaking countries are non-diverse and/or centralized norm from which all others differ. However, we decided to use the referred terminology as this is still widely used in mental health literature across countries, and also in how the target population was framed in the funding agreement of the service under study.

^2^Costa, N., Olson, R., Mescouto, K., Setchell, J., Plage, S., Dune, T., Prasad, R., Suleman, S., and Ng, Z. Y. (under review). Specialist psychosocial mental health support programs for people with diverse language and cultural backgrounds: a critical rapid review. Culture, Medicine and Psychiatry.

^3^We do not presume to know the skin colour of the doctor and patient referenced in this data display. Instead, we approach race and ‘whiteness’ as intersecting, multiple and context-specific positions of privilege (see Ignatiev, 1995), where skin colour is socially constructed through racialised and classed relations.
